# HIV Surveillance in a Large, Community-Based Study: Results from the Pilot Study of Project Accept (HIV Prevention Trials Network 043)

**DOI:** 10.1186/1471-2334-11-251

**Published:** 2011-09-24

**Authors:** Estelle Piwowar-Manning, Agnes Fiamma, Oliver Laeyendecker, Michal Kulich, Deborah Donnell, Greg Szekeres, Laura Robins-Morris, Caroline E Mullis,, Ana Vallari, John Hackett, Timothy D Mastro, Glenda Gray, Linda Richter, Michel W Alexandre, Suwat Chariyalertsak, Alfred Chingono,, Michael Sweat, Thomas Coates, Susan H Eshleman

**Affiliations:** 1 Dept. of Pathology, Johns Hopkins Univ. School of Medicine, Baltimore, MD, USA; 2 Program in Global Health, Univ. of California at Los Angeles, Los Angeles, CA, USA; 3 Dept. of Medicine, Johns Hopkins Univ. School of Medicine, Baltimore, MD, USA; 4 Laboratory of Immunoregulation, NIAID, NIH, Baltimore, MD, USA; 5 Dept. of Probability and Statistics, Faculty of Mathematics and Physics, Charles University in Prague, Czech Republic; 6 Vaccine and Infectious Disease Division, Fred Hutchinson Cancer Research Center, Seattle, WA, USA; 7 Infectious Diseases Research, Abbott Diagnostics, Abbott Park, IL, USA; 8 FHI 360, Durham, NC, USA; 9 Perinatal HIV Research Unit, Chris Hani Baragwanath Hospital/University of the Witwatersrand, Johannesburg, South Africa; 10 Human Sciences Research Council, Durban, South Africa; 11 Department of Microbiology and Immunology, Muhimbili University of Health and Allied Sciences, Dar es Salaam, Tanzania; 12 Research Institute for Health Sciences, Chiang Mai University, Chiang Mai, Thailand; 13 Dept. of Psychiatry, School of Medicine, University of Zimbabwe, Harare, Zimbabwe; 14 Dept. of Psychiatry and Behavioral Sciences, the Medical University of South Carolina, Charleston, SC, USA

## Abstract

**Background:**

Project Accept is a community randomized, controlled trial to evaluate the efficacy of community mobilization, mobile testing, same-day results, and post-test support for the prevention of HIV infection in Thailand, Tanzania, Zimbabwe, and South Africa. We evaluated the accuracy of in-country HIV rapid testing and determined HIV prevalence in the Project Accept pilot study.

**Methods:**

Two HIV rapid tests were performed in parallel in local laboratories. If the first two rapid tests were discordant (one reactive, one non-reactive), a third HIV rapid test or enzyme immunoassay was performed. Samples were designated HIV NEG if the first two tests were non-reactive, HIV DISC if the first two tests were discordant, and HIV POS if the first two tests were reactive. Samples were re-analyzed in the United States using a panel of laboratory tests.

**Results:**

HIV infection status was correctly determined based on-in country testing for 2,236 (99.5%) of 2,247 participants [7 (0.37%) of 1,907 HIV NEG samples were HIV-positive; 2 (0.63%) of 317 HIV POS samples were HIV-negative; 2 (8.3%) of 24 HIV DISC samples were incorrectly identified as HIV-positive based on the in-country tie-breaker test]. HIV prevalence was: Thailand: 0.6%, Tanzania: 5.0%, Zimbabwe 14.7%, Soweto South Africa: 19.4%, Vulindlela, South Africa: 24.4%, (overall prevalence: 14.4%).

**Conclusions:**

In-country testing based on two HIV rapid tests correctly identified the HIV infection status for 99.5% of study participants; most participants with discordant HIV rapid tests were not infected. HIV prevalence varied considerably across the study sites (range: 0.6% to 24.4%).

**Trial Registration:**

ClinicalTrials.gov registry number NCT00203749.

## Background

Project Accept (HIV Prevention Trials Network [HPTN] 043) is a Phase III, community randomized, controlled trial of community mobilization, mobile testing, same-day results, and post-test support for the prevention of HIV infection [1]. The primary objective of Project Accept is to test the hypothesis that communities receiving three years of community-based voluntary counseling and testing (CBVCT) will have significantly lower HIV incidence compared to communities receiving three years of standard clinic-based VCT (SVCT) alone. In Project Accept, 34 communities in Africa (Soweto and Vulindlela, South Africa) [2-4], Tanzania (Kisarawe), and Zimbabwe (Mutoko) [5,6] and 14 communities in Thailand (Chiang Mai) [7-9] were randomized to receive either a CBVCT intervention in addition to SVCT services, or SVCT services alone. The CBVCT intervention has four major components: (1) to make VCT more available in community settings, (2) to engage the community through outreach, (3) to provide post-test support, and (4) to provide real-time feedback to continually refine the intervention. These strategies were designed to change community norms and reduce risk for HIV infection among all community members, irrespective of whether they participated directly in the intervention.

The intervention began in January, 2006 and was completed in March, 2011. Samples are now being collected from approximately 50,000 participants for the post-intervention assessment. In preparation for analysis of the post-intervention samples, a pilot study was performed that involved collection and analysis of post-intervention samples from approximately 2,500 individuals in additional, non-randomized SVCT communities across the five study sites. In this report, we evaluated the accuracy of in-country HIV testing and determined HIV prevalence in the Project Accept pilot study.

## Methods

### Study participants

Participants were selected from all community residents by cluster sampling based on households. Participating households were selected randomly from the list of all households in the community. Informed consent was obtained from the head of a selected household and a list of eligible household members. Eligibility criteria were age between 18 and 32 years and residency in the community. All eligible household members were invited to participate in the pilot study. Written informed consent was obtained from all study participants.

### Sample collection

Blood samples were collected by venous puncture by a trained phlebotomist adhering to approved bio-safety procedures. Two tubes were collected from each participant: a 10 ml EDTA-anticoagulated blood sample (for in-country HIV diagnostic testing and sample storage for testing at the HPTN Network Laboratory) and a 5 ml sample for CD4 cell count testing; note that CD4 cell count test results were not analyzed in this substudy. Each blood sample was assigned a Blood ID number. Tubes were mixed by inverting 5-8 times, following the manufacturer's instructions, immediately after sample collection. Samples were transported to collaborating local testing laboratories in cooler boxes following local regulations for transportation, and were delivered to the testing laboratories within a sufficient time frame to allow processing and freezing within 24 hours of sample collection. EDTA-anticoagulated samples were stored and transported at 4-25°C prior to testing.

### In-country testing

In-country HIV diagnostic testing was performed at collaborating local testing laboratories according to local Ministry of Health guidelines. For each participant, the laboratory performed two HIV rapid tests in parallel. Testing in Thailand, Tanzania and Zimbabwe was performed using whole blood; testing at the two South African sites was performed using plasma. The HIV rapid tests used at each of the study sites are shown in Table 1. If both HIV rapid tests were non-reactive, the participant was considered to be HIV-uninfected; stored samples from these participants were designated HIV NEG. If both HIV rapid tests were reactive, the participant was considered to be HIV-infected; stored samples from these participants were designated HIV POS. If one of the two HIV rapid tests was reactive and one of the two HIV rapid tests was non-reactive (if the HIV rapid tests results were discordant), a third diagnostic test (tie-breaker, either an EIA or a third HIV rapid test) was performed to determine the participant's HIV status; regardless of the results of the third test, samples from these participants were designated HIV DISC. Each study site adhered to standards of Good Clinical Laboratory Practice [10], the HPTN Manual of Laboratory Operations, and local standard operating procedures for proper collection, processing, labeling, transport of specimens to the local testing laboratories, storage, and shipping of specimens to the HPTN Network Laboratory.

**Table 1:** HIV rapid tests and chemiluminescent immunoassays used at study sites*

	Thailand	Tanzania	Zimbabwe	South Africa Vulindlela	South Africa Soweto
Assay #1	Determine HIV 1/2^a^	Determine HIV 1/2^a^	Determine HIV 1/2^a^	Determine HIV 1/2^a^	Determine HIV 1/2^a^

Assay #2	Bioline HIV1/2^a,b^	SD Bioline HIV 1/2 v3^a^	Uni-Gold HIV^a^	SD Bioline HIV 1/2 v3^a^	SD Bioline HIV 1/2 v3^a ^or Uni-Gold HIV^a^

Assay #3 (tie-breaker)	Clearview* HIV 1/2 STAT-PAK^c^	Uni-Gold HIV^a^	OraQuick HIV-1/2 Rapid Antibody Test^c^	ARCHITECT HIV Ag/Ab Combo CMIA^c^	ARCHITECT HIV Ag/Ab Combo CMIA^c^

* The following assays were used in the study: Determine HIV 1/2 (Inverness Medical Innovations, Petchabun, Japan), SD Bioline HIV 1/2 version 3 (Youngin-Si, South Korea), Bioline HIV 1/2 (Petchaboon, Thailand), Uni-Gold HIV (Trinity Biotech plc, Bray, Ireland), Clearview HIV 1/2 STAT-PAK (Inverness Medical Innovations, Waltham, MA), OraQuick HIV 1/2 Rapid Antibody Test (OraSure Technologies, Inc., Bethlehem, PA), ARCHITECT HIV Ag/Ab Combo (Abbott Diagnostics, Wiesbaden, Germany). CMIA: chemiluminescent microparticle immunoassay.

^a ^Recommended by the USAID [*pdf.usaid.gov/pdf_docs/PNADO095.pdf] accessed 11/23/10 - *HIV Test Kits Listed in the USAID Source and Origin Waiver: Procurement Information Document, Fifth Edition, Edited by Abiola Johnson, January 2009].

^b ^Cleared by the Thailand Food and Drug Administration.

^c ^Cleared by the United States Food and Drug Administration.

### Data management

Specimen collection, testing, storage, and shipping were tracked using the Laboratory Data Management System (LDMS). Participant data and laboratory results obtained at the study sites were submitted to the Project Accept Statistical Center, where those data were merged and checked for completeness and consistency. Cleaned data was submitted to the HPTN Statistical and Data Management Center (SCHARP, Seattle, WA).

### Laboratory testing at the HPTN Network Laboratory

Quality control testing was performed in the HIV Specialty Laboratory at the HPTN Network Laboratory at the Johns Hopkins University School of Medicine and Johns Hopkins Hospital (Baltimore, MD); this laboratory is accredited by the College of American Pathologists and certified under the Continuous Laboratory Improvement Act 1988 (CLIA-certified). Testing included the following assays: the Vitros EIA Human Immunodeficiency Virus Type 1 and/or 2 (HIV-1/2) Antibody Detection in Human Serum and Plasma (VITROS ECi/ECiQ Immunodiagnostic System, Ortho Diagnostics, Johnson & Johnson, Pencoed, United Kingdom), Genetics System HIV-1 Western Blot (BioRad Laboratories, Redmond WA), APTIMA^® ^HIV-1 RNA Qualitative Assay (Gen-Probe Inc., San Diego, CA). All assays were performed according to the manufacturers' instructions.

### Laboratory testing at Abbott Diagnostics

Samples designated HIV NEG based on in-country testing were tested using the ARCHITECT HIV Ag/Ab Combo assay (HIV Combo; List: 2P36; Abbott Diagnostics, Wiesbaden, Germany). HIV Combo testing was performed at Abbott Diagnostics (Abbott Park, IL). The HIV Combo assay was performed according to the manufacturers' instructions, with the following exception: Based on the package insert, specimens that are initially reactive in the HIV Combo assay should be retested in duplicate when the assay is used for HIV diagnosis in a clinical setting. In this study, the assay was used only to confirm prior test results for research purposes; the assay was performed only once to minimize the volume of samples used for this testing, and any sample that was reactive on this initial screening test was further evaluated using other assays.

### Informed consent

This research was performed in compliance with the Helsinki Declaration. The Project Accept Pilot study was approved by ethical review committees for each of the Project Accept study sites and collaborating institutions.

## Results

### In-country sample collection and testing

In Project Accept, the study team visited households in the study communities and enrolled eligible participants who consented for the study (Table 2). Overall, 2,452 (76.3%) of 3,212 members of the selected households were eligible for the study; 2,247 (91.6%) of the eligible individuals consented to participate, and had a sample collected by a trained phlebotomist. Overall, 1,906 participants were characterized as HIV-negative (HIV NEG), 24 were characterized as HIV-discordant (HIV DISC), and 317 were characterized as HIV-positive (see Methods, Table 2). A third tie-breaker test was used to define further the infection status of the 24 participants with discordant HIV rapid tests, but the original designation of the participants as HIV DISC was not changed in the study data set.

**Table 2:** Collection and laboratory analysis of samples included in the Project Accept pilot study*

	Thailand	Tanzania	Zimbabwe	Soweto South Africa	Vulindlela South Africa	Subtype C (3 sites)	All five sites
**Prevalent HIV subtype(s)**^a^	CRF01_AE	Multiple	C	C	C		

**In-country sample collection and testing**							

# household members^b^	512	683	663	780	574	2,017	3,212

# eligible participants^c^	348	417	553	625	509	1,687	2,452

# participants with two HIV rapid test results^d^	341	379	504	530	493	1,527	2,247

# HIV NEG samples	336	348	426	430	366	1,222	1,906

# HIV DISC samples	3	13	3	3	2	8	24

# HIV POS samples	2	18	75	97	125	297	317

HIV prevalence^e^	0.6%	4.7%	14.9%	18.3%	25.4%	19.4%	14.1%

**Analysis of HIV NEG samples**							

# HIV Combo reactive^f^		3	1	10		11	14

# confirmed HIV-negative		2	1	4		5	7

# HIV-positive, WB positive				6		6	6

# HIV-positive, WB indeterminate		1					1

**Analysis of HIV DISC samples**							
# HIV-negative^g^	3	13	3	3	1	7	23

# HIV-positive, WB positive^h^					1	1	1

**Analysis of HIV POS samples**							

# HIV-negative^i^			1		1	2	2

**Corrected sample numbers**^j^							

Corrected # HIV NEG samples	339	360	430	427	368	1,225	1,924

Corrected # HIV POS samples	2	19	74	103	125	302	323

Corrected HIV prevalence	0.6%	5.0%	14.7%	19.4%	24.4%	19.8%	14.4%

*WB: Western blot; OD-n: normalized optical density units; POS: positive; DISC: discordant; NEG: negative.

^a^The prevalent HIV subtypes at each site are indicated. Most HIV strains in Thailand are CRF01_AE. Multiple HIV subtypes are prevalent in Tanzania, including HIV subtypes A, C, and D. Most HIV infections in South Africa and Zimbabwe are subtype C. The column labeled "Subtype C" shows combined results from Vulindlela and Soweto, South Africa, and Zimbabwe.

^b^All household members (see Methods)

^c^Number (#) eligible participants excludes participants who had no contact, declined participation, were ineligible for the study, or had missing status.

^d^Samples were characterized based on the results of the two HIV rapid tests (see Methods): HIV POS: two reactive HIV rapid tests. HIV DISC: one reactive and one non-reactive HIV rapid test. HIV NEG: two non-reactive HIV rapid tests.

^e^HIV prevalence based on in-country testing: # HIV POS samples/total # samples × 100.

^f^Fourteen samples were initially reactive with the ARCHITECT HIV Ag/Ab Combo assay (HIV Combo, signal/cutoff >1). Note that according to the package insert, specimens that are initially reactive with HIV Combo must be retested in duplicate and only repeatedly reactive specimens are considered reactive. In this study, samples were analyzed only once because sample volumes were limiting.

^g^Twenty-two samples were non-reactive with the Vitros EIA and non-reactive with the Aptima HIV RNA test; one sample was reactive with the Vitros EIA and non-reactive with the Aptima HIV RNA test.

^h^One sample was reactive with the Vitros EIA and was Western blot positive.

^i^Two samples were non-reactive with the Vitros EIA and non-reactive with the Aptima HIV RNA test.

^j^Corrected sample numbers: The numbers of HIV POS and HIV NEG samples were adjusted by taking into account reclassification of samples based on quality control testing performed at the HPTN Network Laboratory.

### Evaluation of the accuracy of in-country testing

Samples from participants in the Project Accept pilot study were analyzed at the HPTN Network Laboratory to confirm and/or clarify the HIV infection status of each participant. Test results for each participant group (HIV NEG, HIV DISC, and HIV POS) are presented below and in Table 2.

#### Evaluation of HIV NEG samples

Overall, 1,906 samples had two non-reactive HIV rapid test results (HIV NEG, Table 2). Those samples were initially screened using a combined HIV antigen/antibody test, the ARCHITECT HIV Ag/Ab Combo assay (HIV Combo, see Methods). This testing confirmed that 1,893 (99.3%) of the 1,906 samples were HIV-negative; the remaining 14 samples (0.73%) had a reactive HIV Combo test result. Further testing confirmed that six of the 14 samples were HIV-negative (five samples were non-reactive with the Vitros EIA assay and the APTIMA HIV RNA test; one sample was reactive with the Vitros EIA, but had a negative Western blot and was non-reactive with the APTIMA HIV RNA assay; the median HIV Combo result for these six samples was signal/cut-off (s/co) = 3.3, range = 1.3-15.6).

Seven of the remaining eight samples with reactive HIV Combo test results were confirmed to be HIV-positive; six were Western blot positive (all from Soweto, HIV Combo results: median s/co = 438.5, range 395.5-512.2) and one was Western blot indeterminate (from Tanzania, HIV Combo result: s/co = 2.71); one sample could not be evaluated further because there was insufficient plasma remaining for additional testing (HIV Combo result: s/co = 3.72; s/co > 1 is considered to be reactive).

#### Evaluation of HIV DISC samples

Twenty-four samples in the pilot study had discordant HIV rapid test results (HIV DISC, Table 2); the in-country tie-breaker test was reactive for three (12.5%) of those samples. Twenty-two (91.7%) of the 24 samples were non-reactive with the Vitros EIA and were also non-reactive with the Aptima HIV RNA test; one sample was reactive with the Vitros EIA and non-reactive with the Aptima HIV RNA test. Those 23 samples (95.8% of the 24 HIV DISC samples) were considered to be HIV-negative; this includes two samples that had a reactive in-country tie-breaker test and were identified as HIV-infected based on in-country testing (one from Tanzania and one from Vulindlela, South Africa). One sample was reactive with the Vitros EIA and was Western blot positive; this sample had a reactive in-country tie-breaker test and was correctly identified as HIV-positive based on in-country testing.

#### Evaluation of HIV POS samples

Overall, 317 samples in the pilot study had two reactive HIV rapid test results (HIV POS, Table 2). Three-hundred-fifteen (99.4%) of those samples were reactive with the Vitros EIA and were considered to be HIV-positive, confirming the in-country test results. The remaining two samples were non-reactive with the Vitros EIA and were non-reactive with the Aptima HIV RNA test; those samples were considered to be HIV-negative (i.e., false positive in-country test results).

### HIV prevalence

HIV prevalence was calculated as: # HIV POS/total # participants tested × 100% (Table 2). The overall HIV prevalence based on in-country testing was 14.1%. HIV prevalence was adjusted to 14.4% based on testing performed at the HPTN Network Laboratory. HIV prevalence was 0.6% in Thailand, 5.0% in Tanzania, 14.7% in Zimbabwe, 19.4% in Soweto, South Africa, and 24.4% in Vulindlela, South Africa. The overall HIV prevalence at the three study sites with prevalent subtype C HIV infection (Zimbabwe and the two sites in South Africa) was 19.8%.

## Discussion

Population surveillance of HIV infection is critical for monitoring the HIV/AIDS epidemic, identifying individuals in need of HIV care, identifying both HIV-infected and HIV-uninfected individuals for HIV counseling to reduce the risk of HIV transmission, and evaluating interventions for HIV prevention. The widespread availability of HIV rapid tests has facilitated HIV surveillance. HIV rapid testing with two or more assays has also replaced use of Western blots for HIV diagnosis in many countries. In Project Accept, HIV surveillance involved collection of blood samples in communities. At four of the five study sites, blood collection was performed in home-based settings; at one study site (Thailand), the vast majority of blood collection was performed at a community center, with only a small number of samples collected in home-based settings. Samples were transported to local laboratories for testing by trained and certified Medical Technologists. Those laboratories demonstrated successful performance in External Quality Control programs, and were also monitored and audited by the HPTN Network Laboratory, the HPTN Statistical and Data Management Center, the National Institutes of Health (NIH) Division of Allergy and Infectious Diseases (NIAID), and an external monitoring group as part of this HPTN clinical trial. For these reasons, the accuracy of laboratory testing, data and sample management may be higher in this study than in other clinical and field-based HIV surveillance settings (e.g., where HIV rapid testing is performed in a non-laboratory or home-based setting by non-laboratory health workers). However, adding external improvement measures can improve the performance of such HIV rapid testing [11] and multiple rapid test algorithms have been shown to be very accurate when used in diverse African settings [12-14]. In the Project Accept pilot study, the requirement for real-time CD4 cell count testing and sample storage for quality control and other testing at the HPTN Network Laboratory necessitated use of phlebotomy (rather than finger-stick) for all Project Accept participants; the post-intervention assessment phase of Project Accept has similar requirements for sample collection and testing. Data from the pilot study of Project Accept (this report) and the on-going post-intervention assessment (data not shown) demonstrate high rates of acceptance and success of phlebotomy performed in communities, mostly in home-based settings. Among 2,452 eligible participants, 2,299 (93.7%) consented to participate in the Project Accept pilot study; 2,247 (97.7%) of those participants had two HIV rapid tests obtained in-country and had samples stored and shipped to the HPTN Network Laboratory for testing. This demonstrates the feasibility of using this approach for community-based research studies. In the Project Accept pilot study, participants were provided with pre-test counseling and information about how to access their in-country test results. Participants who accessed their test results were provided with post-test counseling, and those with positive test results were referred to treatment centers. However, most participants did not seek their results. In other studies, such as those that include a "test and treat" objective, HIV rapid testing in the field using a finger-stick sample (with or without subsequent phlebotomy for those who are likely to be HIV-infected) would allow for real-time communication of HIV test results and referral for HIV care and treatment.

We detected relatively low rates of false positive and false negative on-site test results; HIV status based on analysis of samples at the HPTN Network Laboratory was different from the HIV status based on in-country testing for 11 (0.5%) of 2,247 study participants. Seven (0.37%) of 1,906 samples identified in-country as HIV NEG were found to be HIV-positive. Those samples were identified by testing the HIV NEG samples with the HIV Combo assay, which is similar in cost to other automated serologic assays used for HIV diagnosis. The HIV Combo assay can detect acute (pre-seroconversion) HIV infection, as well as established (antibody-positive) HIV infection; the sensitivity for detection of acute infection with HIV Combo is similar to typically pooled HIV RNA test methods [15-18]. We did not detect any acute HIV infections among the 1,906 HIV NEG samples screened with the HIV Combo assay. Two (0.63%) of 317 samples identified in-country as HIV POS were found to be HIV-negative. In addition, two of the three participants with discordant HIV rapid tests who had a reactive in-country tie-breaker test and were identified in-country as HIV-infected were found to be HIV-negative based on testing at the HPTN Network Laboratory. It is not possible to determine whether these errors (false negative and false positive HIV results) were due to problems with participant identification/sample labeling, testing errors, or clerical errors at the study sites.

In the Project Accept pilot study, the HIV prevalence across the five study sites based on in-country HIV rapid testing was 14.1%. This was adjusted to 14.4% when test results from the HPTN Network Laboratory were taken into account. The HIV prevalence varied widely across the study sites, from 0.6% in Thailand to 24.4% in Vulindlela, South Africa. The highest HIV prevalence rates were found at the three sites where subtype C is prevalent (Zimbabwe and the two sites in South Africa, overall 19.8%).

## Conclusions

This study provides information on the accuracy of HIV surveillance testing performed in the context of a clinical trial, where testing was performed in local laboratories in Thailand, Tanzania, Zimbabwe and South Africa. In-country testing based on two HIV rapid tests correctly identified the HIV infection status for 99.5% of study participants; most participants with discordant HIV rapid tests were not infected. HIV prevalence varied considerably across the five Project Accept study sites (range: 0.6% to 25.4%). Further studies are needed to assess the accuracy of HIV testing in surveillance and clinical programs where testing is performed in non-laboratory (e.g., home-based) settings. Further research is also needed to identify and validate robust, accurate methods for cross-sectional HIV incidence determination that could easily be incorporated into HIV surveillance programs.

## List of Abbreviations

HIV: human immunodeficiency virus; HPTN: HIV Prevention Trials Network; CBVCT: community-based voluntary counseling and testing; SVCT: standard clinic-based VCT; EIA: enzyme immunoassay; HIV Combo: ARCHITECT HIV Ag/Ab Combo assay; CMIA:. chemiluminescent microparticle immunoassay.

## Competing interests

The authors declare that they have no competing interests, with the following exceptions: A. Vallari and J. Hackett, Jr. are employees and stockholders of Abbott Laboratories, manufacturer of the HIV Combo assay. Dr. Eshleman received an honorarium in 2009 for an invited presentation at a symposium sponsored by Abbott Laboratories.

## Authors' contributions

All authors contributed to the study and to the preparation of the manuscript. All authors reviewed the final version of the manuscript prior to submission. In addition, the authors had the following roles in the project. EPM: Was responsible for overseeing laboratory testing in Project Accept. AF: Is the Laboratory and Operations Study Coordinator for Project Accept. OL: Contributed to study design and data analysis. MK: Is the Lead Statistician for Project Accept. DD: Is the HPTN Statistician for Project Accept. GS: Is the Multi-site Trial Coordinator for Project Accept. LRM: Was the HPTN data analyst for Project Accept. CEM: Performed testing with the BED and avidity assays. AV: Coordinated testing with the COMBO HIV Ag/Ab assay and reviewed test results. JH: Coordinated for testing with the COMBO HIV Ag/Ab assay. TDM: Reviewed literature related to use of HIV rapid testing in surveillance studies. GG: Is the Principal Investigator for the Soweto, South Africa site, Project Accept. LR: Is the Principal Investigator for the Vulindlela, South Africa site, Project Accept. MA: Is the Laboratory QA/QC Coordinator, Muhimbili University of Health and Allied Sciences, Tanzania site, Project Accept. SC: Is the Principal Investigator for the Chiang Mai, Thailand site, Project Accept. AC: Is the Principal Investigator for the Mutoko, Zimbabwe site, Project Accept. MS: Is the Chair of Utilization and Cost-Effectiveness Cores for Project Accept and the U.S.-based Principal Investigator for the Tanzania site. TC: Is the Steering Committee Chair for Project Accept and the U.S.-based Principal Investigator for the South African sites. SHE: Conceived of the study, designed the study, analyzed the data, drafted and finalized the manuscript.

## Pre-publication history

The pre-publication history for this paper can be accessed here:

http://www.biomedcentral.com/1471-2334/11/251/prepub
